# An unusual cause of granulomatous colitis: Behcet's disease

**DOI:** 10.1002/jgh3.12215

**Published:** 2019-08-15

**Authors:** Ashutosh I Yadav, Arghya Chattopadhyay, Rizwan Ahamed, Gaurav Muktesh, Tarun Narang, Ritambhra Nada, Aman Sharma, Harshal Mandavdhare, Vishal Sharma, Usha Dutta

**Affiliations:** ^1^ Department of Internal Medicine Postgraduate Institute of Medical Education and Research Chandigarh India; ^2^ Department of Gastroenterology Postgraduate Institute of Medical Education and Research Chandigarh India; ^3^ Department of Dermatology Postgraduate Institute of Medical Education and Research Chandigarh India; ^4^ Department of Histopathology Postgraduate Institute of Medical Education and Research Chandigarh India

**Keywords:** clinical, intestinal disorders, inflammatory bowel diseases, intestinal disorders

## Abstract

Intestinal involvement in Behcet's disease (BD) is uncommon. Differentiating it from close mimics like Crohn's Disease is difficult. Further, in asymptomatic cases, diagnostic challenges posed is still greater. A 17‐year‐old boy had history of recurrent oro‐genital ulcers, papulo‐pustular skin eruptions, ocular lesions and had presented with recent onset perianal abscess. Fecal calprotectin was elevated, and ileo‐colonoscopy showed ileocolonic ulcers of inflammatory nature. Clinical diagnosis of intestinal BD was made. Patients with BD having mucosal lesions may harbor asymptomatic intestinal lesions. Screening them with fecal calprotectin levels and if positive, with subsequent imaging and endoscopic biopsy with timely initiation of appropriate treatment in such asymptomatic cases help to control overall disease activity.

## Graphical abstract

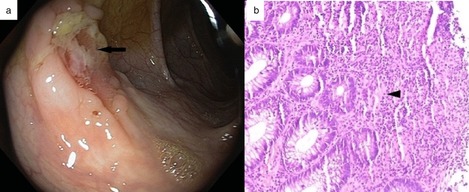

Many diseases can cause intestinal granulomas like Crohn's disease and tuberculosis. Behcet's disease is an uncommon cause of intestinal granulomas. Here, we present the case of intestinal Behcet's that closely mimicked Crohn's disease.

## Introduction

Behcet's disease (BD) is a chronic, multisystem, inflammatory disorder with an underlying vasculitic process. It shares many common features with Crohn's disease (CD), and these have even been considered two sides of the same coin.^1^ Differentiating between the two is important because the management of BD, although similar to CD, is not essentially the same.^2^, ^3^ BD with no gastrointestinal complaints may suggest pathological evidence of gut inflammation preceding clinical manifestation. While CD is a well‐recognized cause of granulomatous colitis, BD is an uncommon cause of granulomatous colitis.

## Case report

**Figure 1:**
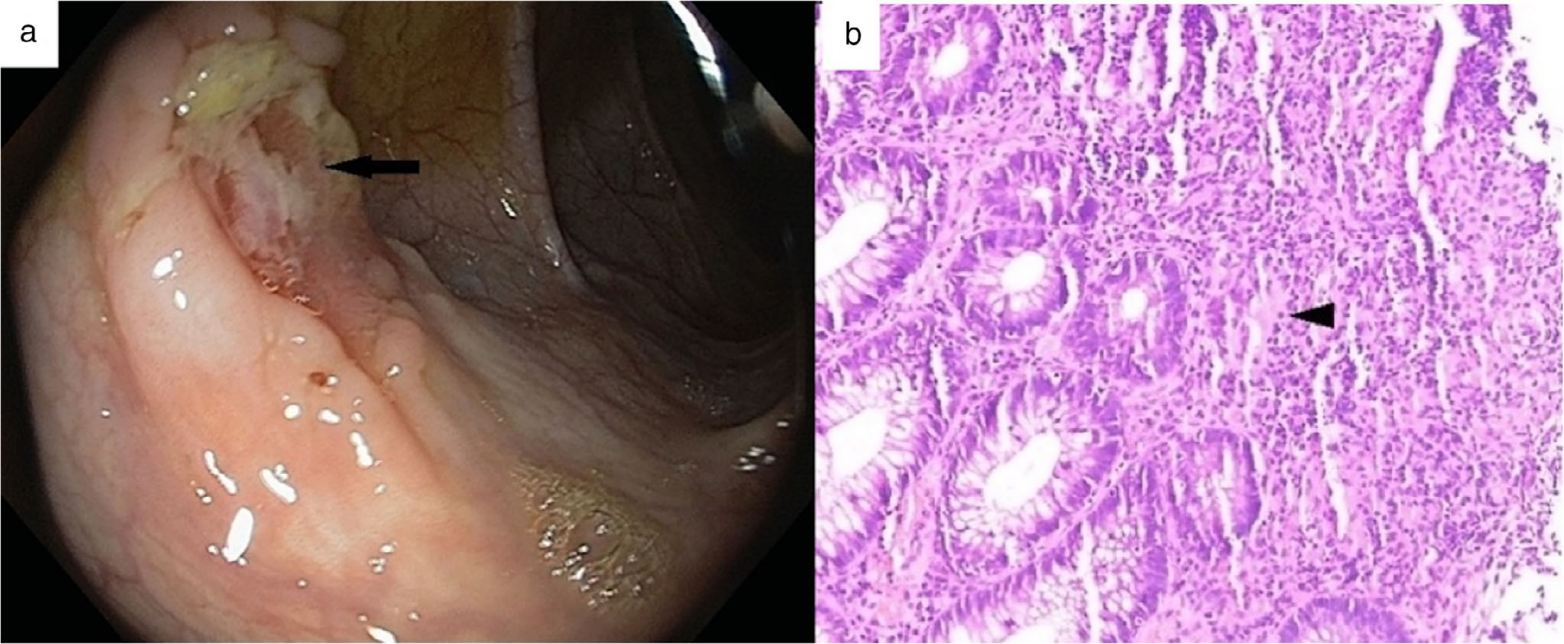
(a) Colonoscopy image showing a typical ulcer of intestinal Behcet's disease, which is round to oval shaped with a discrete margin (black arrow). (b) Histopathological microphotograph (H&E stain) of colon (20× original magnification) showing chronic colitis with a small, submucosal, noncaseating, ill‐defined, epithelioid granuloma (black arrow head).

A 17‐year‐old boy presented to our department with recurrent orogenital ulcers, skin lesions in the form of papulopustular eruptions, and conjunctival erosions for 1 year with recent‐onset perianal abscess for 1 month. He was diagnosed with BD as he fulfilled both the International Study Group (ISG) criteria and International Criteria for Behcet's Disease (ICBD) (recurrent genital ulceration and eye lesions: 2 points each and recurrent oral ulceration and skin lesions: 1 point each; total points: 6) and was started on colchicine for skin lesions. Routine investigation showed leukocytosis along with elevated C‐reactive protein (CRP) levels (117 mg/L [*n*: 0–10 mg/L]), while HLA‐B5 (51/52) was negative. Perianal abscess was drained. To rule out the possibility of inflammatory bowel disease, fecal calprotectin levels were measured, which was elevated (187.41 μg/gm [*n* < 43.2 μg/gm]). Computed tomogrpahy (CT)‐enterography and ileocolonoscopy with biopsy were performed. CT showed no evidence of mural thickening, strictures, fistulae, obstruction, or dilatation. Ileocolonoscopy showed multiple, round to oval, ulcers varying in size from 0.5–1 cm^2^ throughout the ileum, caecum, ascending colon, transverse colon, descending colon, and rectosigmoid (Fig. 1a). Colonoscopic biopsy showed evidence of active inflammation along with features of chronicity in the form of crypt distortion, crypt loss, and Paneth cell metaplasia; occasional ill‐defined epithelioid cell granulomas; and submucosal ischemia with evidence of fibrosis (Fig. 1b). Stains for fungal element and acid‐fast bacilli were negative, along with negative polymerase chain reaction (PCR) for *Mycobacterium tuberculosis*. The patient was started on oral mesalazine [1 g thrice daily (TDS)] along with ω‐3 fatty acid, oral zinc (40 mg elemental Zn), and multivitamin supplementation.

The patient was followed up for 2 months. His skin lesions and oral lesions had healed, and repeat ileocolonoscopy showed mucosal healing with resolution of most of the ulcers and few scattered healed lesions in the ileum and colon. Perianal fistula was healing, with the presence of minimal discharge. Follow‐up CRP levels showed a decreasing trend (69.4 mg/L), while fecal calprotectin was near normal (45.10 μg/gm).

## Discussion

BD is classified as variable vessel vasculitis and can involve vessels of any size (small, medium, and large) and type (arteries, veins, and capillaries).^4^ The criteria used to classify a disease as BD include ISG‐1990^5^ and, more recently, ICBD‐2006.^6^ Recurrent oral ulceration was mandatory for making a diagnosis of BD according to the ISG criteria for BD and International Criteria for Behcet's Disease (ISBD) criteria; however, in ICBD classification, vascular manifestations are included and based on points given to each manifestation, and a diagnosis of BD is made if the total points are ≥3.

BD involves multiple systems, and gastrointestinal involvement may contribute to significant morbidity. In patients with genetic susceptibility (HLA B‐51, single nucleotide polymorphism in IL‐10, IL‐23R/IL‐12RB2 genes etc.), trigger factors (bacteria, virus, or protein particle exhibiting molecular mimicry) precipitates perturbation in T‐cell homeostasis with predominant Th‐1‐mediated inflammatory response, unlike CD where the response is predominantly Th‐17‐mediated, leading to the characteristic manifestation of BD.

Ileocecal involvement is typically more common in the intestinal form of BD as seen in patients with CD or intestinal tuberculosis, but any site from the oral cavity to rectum may be involved. Intestinal manifestations may either be due to neutrophilic phlebitis or large vessel disease. Predominant ulcerative lesions are seen, which are characteristically oval to round, and are deep with a discrete border in the ileocecal region; however, focal, segmental, or diffuse involvement can also be present. In intestinal BD, the most common manifestation is abdominal pain. Other presentations include diarrhea, fever, fistula, or—rarely—acute abdomen in the form of perforation and gastrointestinal bleeding. However, our index patient was asymptomatic.

A diagnosis of BD with intestinal involvement requires a multimodality approach, with the most important being an ileocolonoscopy to identify the presence of a typical ulcer with characteristic features. As described by Cheon *et al*.,^7^ a case of systemic BD with the presence of a typical intestinal ulcer is diagnosed as a case of definite intestinal BD. Laboratory investigations, such as CRP levels and fecal calprotectin, predict active disease with the evidence of gastrointestinal involvement and guide the need for an active search for evidence of intestinal BD even in asymptomatic cases. Serological markers like anti‐Saccharomyces cerevisiae antibodies (ASCA) do not help in differentiating BD from CD due to similar positivity rates. HLA B51 has a negative association with intestinal involvement in BD. In patients with normal ileocolonoscopy but a high possibility of intestinal involvement, capsule endoscopy may be performed to evaluate small bowel involvement. Characteristically, the histopathological finding of vasculitis in the form of ischemic changes and submucosal fibrosis leads to the diagnosis of intestinal BD. However, due to a lack of availability of deeper tissue during endoscopic biopsy and the patchy nature of disease, most of the time, there is only evidence of chronic, nonspecific mucosal inflammation, which makes it difficult to differentiate from CD.^8^

5‐ASA compounds are the first‐line treatment in patients with mild intestinal BD, which can be used for induction and maintenance therapy, in contrast to CD, where the first‐line therapy is steroids and immunosuppression. However, in unresponsive cases and those with moderate to severe disease, a combination of 5‐ASA and oral steroids is used for the induction of remission, followed by 5‐ASA for maintenance. In unresponsive patients, anti‐tumour necrosis factor (TNF) agents, thalidomide, and immunomodulators (e.g. azathioprine) can be used. Surgery is reserved for patients who are refractory to medical therapy or have presented to the emergency department with severe gastrointestinal bleed or perforation.

It is very important to differentiate intestinal BD from CD. Despite considerable overlap in the presentation of both diseases, the management approach, complications, and disease outcomes are likely to be different.^9^ The diagnostic dilemma that we faced in this patient was to differentiate between intestinal BD and CD as the case showed considerable overlap of symptoms and signs. However, the presence of genital ulcers, especially scarring penile and scrotal ulcers and papulopustular skin lesions, favored BD. Although perianal fistulas are more common in CD, there was no radiological feature of CD, and on colonoscopy, a typical intestinal ulcer characteristic of BD was seen without any feature of internal fistulation, strictures, or cobblestone. Although the colonoscopic biopsy did not demonstrate any definite evidence of vasculitis, evidence of chronic inflammation with submucosal ischemia and fibrosis was suggestive of intestinal BD. However, we should be aware that intestinal BD may be missed in a superficial colonoscopic biopsy as sufficient tissue depth may not always be achieved to biopsy the involved vessels. The most striking character in this case was that the patient never had any abdominal complaints, and elevated fecal calprotectin levels provided a clue to underlying mucosal inflammation, which subsequently completely responded to 5‐ASA compounds without any need to add oral steroids, unlike ileocaecal CD.^10^

In conclusion, this case illustrates the importance of suspecting intestinal disease in patients with BD who present with mucosal lesions, screening them for fecal calprotectin levels, and—if positive—following that with subsequent imaging and endoscopic biopsy with timely initiation of appropriate treatment in such asymptomatic cases.
